# Development of Montanide-based inactivated vaccine against duck plague virus in Bangladesh

**DOI:** 10.5455/javar.2025.l967

**Published:** 2025-09-28

**Authors:** Layla Yasmin, Md. Juwel Hossain, Towhidul Islam, Mohammad Ferdousur Rahman Khan, Marzia Rahman, Tazrin Kamal, K. H. M. Nazmul Hussain Nazir, Md. Tanvir Rahman, Rony Ahmed, Mohammad H. Rahman, Md. Bahanur Rahman

**Affiliations:** Department of Microbiology and Hygiene, Bangladesh Agricultural University, Mymensingh, Bangladesh; † These authors are equally contributed.

**Keywords:** DPV, ELISA, efficacy, inactivated DP vaccine, montanide, potency

## Abstract

**Objective:**

This work aimed to develop Montanide-based inactivated duck plague (DP) vaccines from field isolates in Bangladesh and to evaluate the safety, potency, and efficacy.

**Materials and Methods:**

Suspected DP samples such as liver, spleen, trachea, and so on (*N *= 211) were collected from Netrokona, Mymensingh, and Kishoreganj districts. Duck plague virus (DPV) was identified through PCR and characterized by partial sequencing. Following pathogenicity tests in ducklings, the vaccine candidate virus was propagated in embryonated duck eggs and inactivated with 0.2% formalin to formulate 45% Montanide ISA 78 VG and ISA 71 VG-based vaccines. Formulated vaccines were administered following safety tests to G1 and G2, whereas G3 received 1X phosphate buffer saline. Blood samples were collected, and antibody titers were measured using an ELISA kit for up to 6 months. A challenge study was conducted to determine the potency of vaccines.

**Results:**

The prevalence rate was 65.40% (138/211) of DPV-suspected samples, where Netrokona, Mymensingh, and Kishoreganj were 67.81% (59/87), 64.61% (42/65), and 62.71% (37/59), respectively. The pathogenicity test revealed significant morbidity and mortality in ducklings. Two formulated vaccines comply with the safety criteria in ducklings. In the challenge study, both vaccinated groups (G-1, G-2) achieved 88.89% protection against the virulent DP virus, whereas the control group exhibited 93.33% mortality. The antibody titer measured by ELISA peaked at 21 days and remained till 180 days post-vaccination, which showed a 0.1% (*p *< 0.001) level of significance.

**Conclusion:**

After 6 months of vaccination, the Montanide ISA 78 VG-based vaccine showed slightly higher immunogenicity than ISA 71, though both were demonstrated to be safe against the DP virus.

## Introduction

Duck farming is an important occupation in the rural areas of Bangladesh, which is contributing significantly to the economic development and food security. The geographical and climatic conditions of Bangladesh, particularly its vast wetlands, floodplains, and numerous water bodies, are an ideal environment for duck farming [1,2].

In Bangladesh, ducks cover up around 17.22% (68.26 million) of the entire poultry population (396.38 million), ranking second, followed by chickens, in table egg production [3]. However, nearly every year in Bangladesh, this crucial sector of poultry farming is severely impacted by the outbreaks of different infectious diseases in the farms [2,4]. Duck plague (DP) ranks among the most subversive diseases with high fatality among those affecting ducks [5]. The infection is commonly known as duck viral enteritis. The causative agent is Anatid alphaherpesvirus 1, which belongs to the Herpesviridae family [6]. It is an enveloped virus characterized by a dsDNA genome, comprising approximately 158,091–162,175 bp in length, encoding 76 distinct genes [5,7,8]. Viral surface glycoproteins interact with host cells, playing a significant role in pathogenesis [9,10].

Ducks generally exhibit greater resistance to infectious diseases compared to chickens, but they remain highly susceptible to Duck plague virus (DPV) [11]. The virus is extremely transmissible and can occur through both direct contact with infected ducks and indirect exposure to contaminated water, feed, litter, and other environmental sources [12].

It can replicate in various hosts and cell types, including ducklings, adult ducks, avian embryos, avian fibroblast cells, kidney cells, and liver cells. Various diagnostic methods are employed to identify this virus, including passive hemagglutination, virus neutralization, culturing on duck embryo fibroblast (DEF) cells, inoculating in ducklings or adult ducks, and molecular confirmation through PCR [4,13,14].

The infection caused by DPV results in notable economic losses, emphasizing the need for the development of effective vaccines as a primary strategy to control and prevent future outbreaks. Two main types of DP vaccines are used: live attenuated, derived from weakened DP viruses, which provide strong protection by inducing a cell-mediated immune response, and inactivated vaccines, which contain killed viruses, trigger an antibody-mediated immune response, but require an additional dose for long-term immunity [15–19]. Inactivated vaccines have demonstrated higher protective efficacy compared to live attenuated vaccines. Therefore, an inactivated vaccine might be a potential alternative to the live attenuated duck plague vaccines [2,12].

In Europe and the USA, both attenuated and inactivated DP vaccines are used to control the duck plague infection [20,21]. In Bangladesh, the attenuated DP vaccine manufactured by the Livestock Research Institute has occasionally been shown to provide inadequate protection against the wild-type viruses due to the antigenic mismatch with the vaccine strain [2]. Despite DPV being a singular antigenic virus, the underlying causes of vaccination ineffectiveness and elevated mortality rates in Bangladesh remain ambiguous [22]. The molecular research performed in the *haor* regions revealed that field isolates constitute discrete clusters separate from the vaccine strain in phylogenetic trees, indicating a significant lack of close genetic or antigenic similarity [23]. Epidemiological surveys in Sylhet, Bangladesh, indicated that, despite immunization efforts, DP outbreaks persist, implying variability in the level of protection [24].

Given these challenges, the development of an efficient inactivated DP vaccine for the prevention and control of DP on an urgent basis requires the detection, isolation, and molecular characterization of local DPV. The present study aimed to develop a potential vaccine seed from locally circulating DPV isolates, which were used to formulate effective Montanide-based inactivated DP vaccines.

## Materials and Methods

### Ethical approval

The potency evaluation of the DP vaccine and subsequent challenge study were carried out using the virulent DP virus in Jinding ducks, following the ethical guidelines set by the Animal Welfare and Experimental Ethics Committee of Bangladesh Agricultural University (BAU), Mymensingh, Bangladesh, under approval number AWEEC/BAU/2023, dated April 4, 2023.

### Sample collection

A total of (*N *= 211) samples suspected of DP infection were collected from different affected duck farms in Netrokona (*N *= 87; 24°87’ N latitude, 90°80’ E longitude), Kishoreganj (*N* = 59; 24°21’ N latitude, 90°95’ E longitude), and Mymensingh Sadar (*N* = 65; 24°74’ N latitude, 90°37’ E longitude) districts of Bangladesh. Necropsy was performed on ducks suspected of DPV infection, and visceral organs, including liver, spleen, trachea, esophagus, proventriculus, and intestine, were placed in 50 ml Falcon tubes containing 20 ml sterile virus transport medium. Samples were transported in a cool box with ice packs to the Laboratory of the Department of Microbiology and Hygiene, BAU, Bangladesh. Samples were stored at −20°C in the Virology laboratory for subsequent analysis.

### Inoculum preparation

Tissue samples of liver, spleen, trachea, esophagus, proventriculus, and intestine from suspected ducks were homogenized with sterile sea sand to prepare a 10% sample suspension with 1X phosphate buffer saline (PBS) [14]. Centrifugation of the viral suspension was performed at 7,000 rpm for 10 min at 4°C, and the supernatant was collected and filtered through a 0.45 µm Sartorius syringe filter (Thermo Scientific, Germany) [25]. The supernatant fluids were treated with Gentamycin at 100 µl/ml for 2 h at ambient temperature, and 100 µl of fluid after treatment was inoculated in bacteriological and fungal media for sterility tests. Sterile fluid samples were kept at −20°C, which were later used for DPV isolation and further analysis.

### Molecular detection of DPV by PCR

A DNA extraction kit (Promega, USA) was used to extract viral DNA from the samples. DPV was identified by PCR using specific primers of the DNA polymerase and *UL* gene. PCR products were amplified using specific primers for the DNA polymerase gene: F: 5^�^-GAA GGC GGG TAT GTA ATG TA-3^�^, R: 5^�^-CAA GGC TCT ATT CGG TAA TG-3^�^, and *UL* gene: 5^�^-GGC TGG TAT GCG TGA CAT-3^�^, R: 5^�^-GTA TTG GTT TCT GAG TTG GC-3^�^ [4,10,25]. For amplification of target genes, 25 µl of PCR mixture was prepared containing 12.5 µl of Taq 2x PCR mix with dye V2 (ABclonal, USA), 2 µl of primers, 5.5 µl of nuclease-free water (Promega-Madison, USA), and 5 µl (207 ng/µl) of extracted DPV DNA template. The thermal profiles used for both DNA Polymerase and the *UL* gene were 94°C for 2 min; 30 cycles of 94°C for 1 min; 56°C for 1 min; 72°C for 2 min; and a final extension at 72°C for 7 min [4,25].

After completion of the PCR, a 1.5% agarose gel was used to separate amplicons, and DNA bands were visualized under a UV-transilluminator (Bio-Rad, Germany) that was stained with Midori Green Advance Safe DNA dye (Nippon Genetics, Europe) [1,4].

### Partial sequencing and phylogenetic tree analysis

PCR-positive products were delivered to Macrogen Technology Ltd., South Korea, for partial sequencing. A CodonCode Aligner (v12.0.1) was used for data analysis and multiple sequence alignments. The nucleotide sequence data were submitted to GenBank under the accession number PP996337. BLAST was used to compare sequences in the GenBank, and to determine evolutionary relationships; a phylogenetic tree was built using the Neighbor-Joining method using MEGA v12 [26].

### Virus propagation and isolation

To isolate DPV, the prepared inoculum (0.2 ml) was injected via the chorioallantoic membrane (CAM) route of 12-day-old embryonated duck eggs, while control embryonated eggs received 0.2 ml 1X PBS, and the process was continued for up to ten serial passages. Eggs were incubated at 37°C, and embryos that died within 24 h post-inoculation were excluded as nonspecific mortality. After post-infection of 6–7 days, surviving as well as dead embryos were chilled overnight at 4°C [13,14,27]. Then CAMs were collected, processed, centrifuged, and the inoculum was prepared and preserved at −80°C for further study.

DPV isolation was performed in DEF cells prepared from 10-day-old embryonated duck eggs. The DEF cells were maintained in 25 cm^2^ cell culture flasks with minimal essential media (MEM) containing 10% fetal bovine serum (FBS) (Gibco, Grand Island, NY, USA) and an antibiotic-antimycotic solution. Culture flasks were placed in a CO₂ incubator at 37°C for 24 h of incubation [4,28]. Following 24 h of incubation, cell growth was observed under a Carl Zeiss (Germany) inverted microscope. A 1 ml aliquot of CAM suspension was inoculated onto confluent cell monolayers cultured in MEM containing 3% FBS and incubated for 2–3 days. Flasks were observed for cytopathic effect (CPE) after 48–72 h, and flasks that exhibited maximum CPE due to DPV infection were collected and stored at −80°C [28].

### EID_50_ determination

Viral CAM and cell suspensions were serially diluted from 10⁻¹ to 10⁻¹⁰, and for each dilution, five eggs (12-day-old embryos) were inoculated with 0.2 ml through the CAM route. The infection pattern was documented over a 5-day incubation period at 37°C, and the calculation of the EID_50_ was performed using the Reed and Munch method [29].

### Pathogenicity tests

To test the pathogenic potential of the virus, 0.5 ml of CAM suspension containing EID_50_ 10^7.7^/ml of DPV was inoculated intramuscularly in day-old ducklings (*N *= 10) and adult ducks (*N* = 10). Two ducklings and two ducks were injected with an equal volume of 1X PBS and maintained as controls in separate locations. Ducklings and ducks were observed for 14 days for any signs and symptoms of DP [30,31]. Birds showing symptoms of DP were euthanized for subsequent investigation.

### Inactivation of DPV

The DPV BRMH109 isolate was selected as the primary vaccine seed candidate, and it was subsequently used for mass antigen production. DPV antigens were treated with 0.2% formalin and incubated at 37°C for 24 h in a shaker incubator to ensure complete inactivation of the virus [11,32].

### Sterility test

Inactivated DPV antigens and experimental virus suspensions were inoculated in tryptone soya broth (TSB) and blood agar media to ensure sterility from bacterial and fungal contamination [4,14].

### Water in the oil-based DPV inactivated vaccine preparation

Two types of water-in-oil (W/O) emulsion vaccines were formulated using Montanide (SEPPIC Co., France) ISA 78 VG and ISA 71 VG with a ratio of 55:45 [15,20]. The vaccine was properly mixed by the magnetic stirrer. The vaccine dose (0.5 ml of inactivated vaccine/bird) was formulated with the antigen content of EID_50_ 10^7.7^/ml.

### Stability test

A part of the 10 ml antigen used for the vaccine preparation was kept at −20°C for the stability study. The EID_50_ of the antigen was determined at 3-month intervals up to 6 months. After formulation of duck plague inactivated vaccines with Montanide ISA 71 VG and ISA 78 VG, one bottle (100 ml/bottle) of each vaccine was kept at 4°C for 3 months to assess the stability for phase separation, changes in appearance, and viscosity.

### Experimental design of safety, trial, and challenge tests

A total of 290, 1-day-old, unvaccinated Jinding ducklings with no prior infection with duck plague virus were obtained from a duck farm. The ducklings were reared with standard feeding and management requirements with appropriate biosecurity measures. Two hundred forty-five ducklings were allocated randomly into three experimental groups (G-1, G-2, and G-3) for the vaccine trial. The remaining 25 ducklings were grouped for the safety test, and 20 ducklings for the pathogenicity test.

### Safety test

Five groups (5) of ducklings were selected for the safety test. All the ducklings were observed for 7–14 days for any clinical manifestations of DPV.

G-A: day-old duckling injected i/m with 0.5 ml Montanide ISA 78 VG 45% inactivated DP vaccine

G-B: 3-week-old ducklings injected i/m, 1 ml Montanide ISA 78 VG 45% inactivated DP vaccine

G-C: day-old duckling injected i/m with 0.5 ml Montanide ISA 71 VG 45% inactivated DP vaccine

G-D: 3-week-old ducklings injected i/m with 1 ml of Montanide ISA 71 VG 45% inactivated DP vaccine

G-E: Negative Control 

### Experimental vaccine trial

For the vaccine trial, 245 Jinding ducklings were reared in the experimental duck shed in BAU. Before vaccination, blood samples were collected randomly from the experimental groups. At 3 weeks of age, G1 and G2 were injected with the two formulated water-in-oil-based vaccines, and G3 received 1X PBS as a control group. A booster vaccination was administered 30 days after the primary vaccination.

Group 1 (*N* = 100): 0.5 ml (i/m) with Montanide ISA 78 VG inactivated DPV vaccine.

Group 2 (*N* = 100): 0.5 ml (i/m) with Montanide ISA 71 VG inactivated DPV vaccine.

Group 3 (*N* = 45): 0.5 ml (i/m) with 1X sterile PBS.

Blood sera were collected at 14, 21, 28, 60, 90, 120, 150, and 180 days post-vaccination and stored at −20°C for subsequent analysis.

### Antibody titer determined by ELISA

Detection of antibody titer was performed by using the Duck DPV-Ab ELISA KIT (ESEBIO, China) following the manufacturer’s instructions. The cut-off value for ELISA was determined based on optical density (OD) measurement at a wavelength of 450 nm. Cut-off value (OD value) = Average value of negative control wells + 0.15. Duck serum samples were seropositive if their OD value was above the cut-off value and seronegative if it was below [33].

### Vaccine challenge test

For the challenge study, ducks were selected randomly from two vaccinated groups (G-1 = 45 and G-2 = 45) and a negative control group (G-3 = 45). Each duck was inoculated intramuscularly with 1 ml of wild-type duck plague virus at a concentration of EID_50_ 10^7.7^/ml at 21 days post-booster vaccination and observed for 7–14 days for the appearance of clinical signs indicative of DPV infection [14,32].

### Statistical analysis

Antibody titer levels were presented as the mean with standard error. Antibody levels among groups were analyzed using one-way ANOVA with Tukey’s method for multiple comparisons among groups in SPSS software v25 (IBM-SPSS Inc., NY, USA). Data is considered statistically significant if the *p*-value is less than 0.05.

## Result and Discussion

Initial identification of DPV infection in the sample was done by observing clinical signs and symptoms. Suspected samples were further confirmed by a molecular approach.

PCR identification using gene-specific primers confirmed the infection rates across all three suspected districts. Netrokona recorded the highest number of positive samples (59/87); the prevalence rate was 67.81%. Mymensingh and Kishoreganj also demonstrated prevalence rates of 64.61% (42/65) and 62.71% (37/59), respectively. The overall prevalence rate of 65.40% (138/211) exhibited the widespread occurrence of DPV in the study area as described by Soma et al. [11] and Ahamed et al. [13]. The molecular identification of the local isolates involved targeting multiple conserved DPV genes, including DNA polymerase and *UL* gene, consistent with approaches used in previous studies [1,4,13,25].

**Figure 1.**
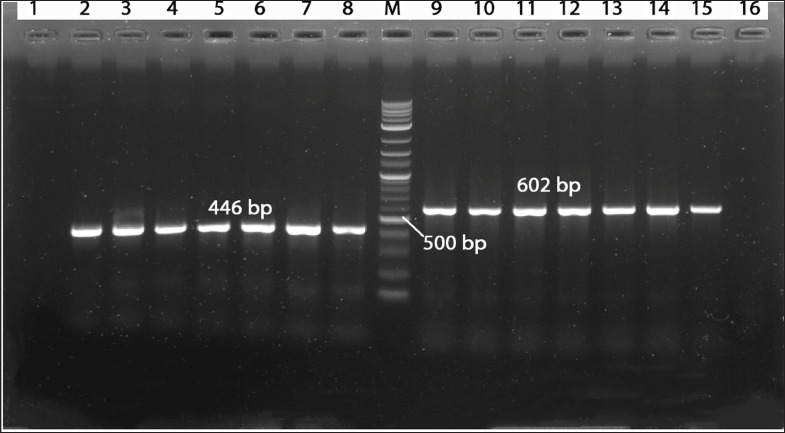
Agarose gel electrophoresis of amplified products for DNA Pol & *UL* gene in DPV. Lane M: 1 kb plus ladder; Lanes 2–7: DPV positive DNA Pol gene at 446 bp; and lanes 10–15: DPV positive *UL* gene at 602 bp. Lanes 8 & 9: Positive control; Lanes 1 & 16: Negative control.

PCR amplification yielded expected DNA fragment sizes of 446 bp for the DNA polymerase gene and 602 bp for the UL30 gene of DPV, as illustrated in Figure 1. These results are consistent with previous studies, where similar gene targets were used for molecular identification of DPV [4,13,25]. DPV prevalence was found to be higher in the district of Netrokona, as per the study by Ahamed et al. [13]. Detection of DPV was confirmed by sequencing and phylogenetic analysis. Partial sequencing of DPV isolate BRMH109 (Accession number PP996337) revealed that this isolate has a common ancestral origin with previously reported strains from Bangladesh, India, China, and the USA (Fig. 4).

**Figure 2.**
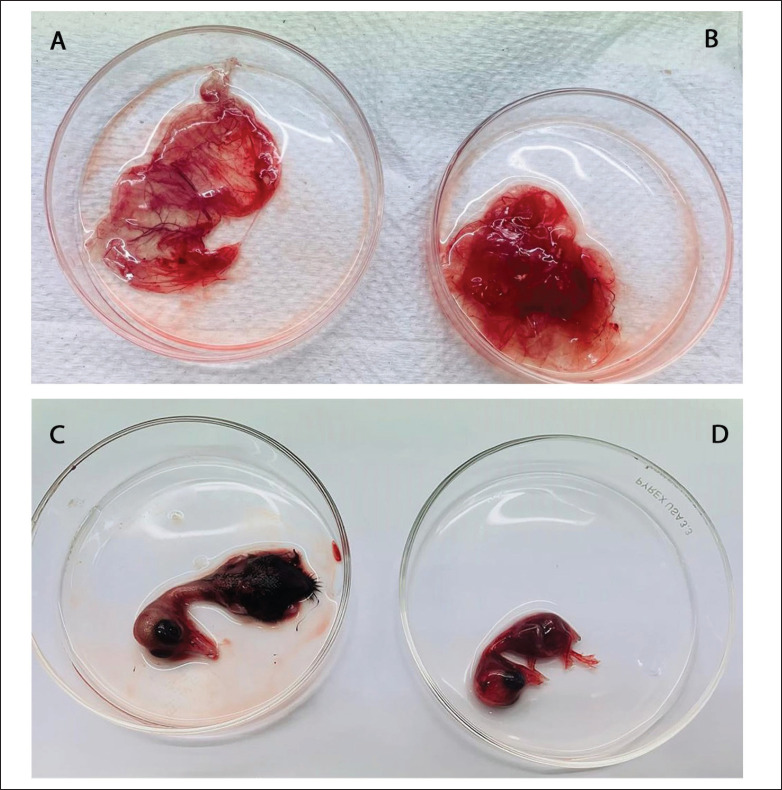
Propagation in the Duck embryo. (A) Non-infected CAM. (B) Hemorrhagic thickened CAM of the infected embryo. (C) Non-infected embryo. (D) Hemorrhagic embryo and underdeveloped plumage after infection with DPV.

DPV infection was evident in the duck embryo when PCR-positive samples were inoculated. We observed embryo mortality at 4–5 days of sample inoculation as described by Ahamed et al. [13] and embryos exhibited similar DPV-specific infections. Infection in the embryo is characterized by subcutaneous hemorrhages, underdeveloped plumage, and thickened CAM, found on gross clinical observation, which were similar to the previous study [11,13]. The corresponding findings are illustrated in Figure 2.

**Figure 3.**
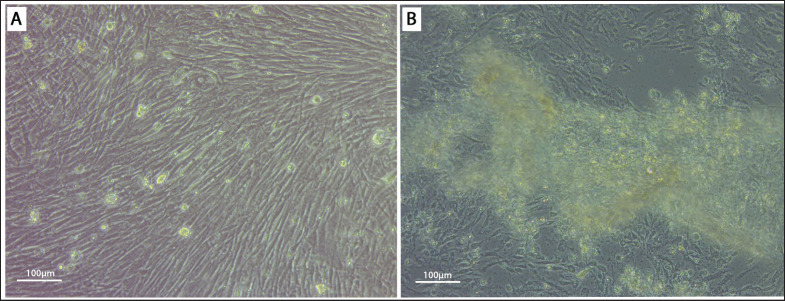
Results of propagation of DPV into duck embryo fibroblast cell culture. (A) Control: showing spindle-shaped fibroblast cells growing together confluently (20x Magnification); (B) DPV-infected: DEF cell after 72 h post-infection. (20x Magnification).

In the DEF cell line, after 48–72 h of virus infection, microscopic observation of DPV-adapted DEF cells showed 90% of the cell death and detachment from the cell culture plate as described by Jahan et al. [4]. CPE was characterized by enlarged, rounded, syncytium-formed, multinucleated giant cell formation and clumped and degenerated fibroblasts, which were consistent with the findings presented in Figure 3 [34].

DPV infection in the egg embryo and DEF cells was confirmed by PCR. The CAM route of the Duck egg embryo was found to be highly susceptible to DPV isolation in the study and holds potential for viral antigen production [27].

The infectivity titer (EID_50_) of the virus from the CAM passaged (10) suspension was calculated as 10^7.7 ^EID_50_/ml. After 5–8 days of virus inoculation, test ducklings showed clinical symptoms of DPV infection, including nervous signs such as tremors of the head, neck, and body; a lowered head; inability to walk; loss of appetite; ataxia; diarrhea; and ultimately death, while the control group remained healthy. The test ducklings were monitored for 14 days. During the observation period, morbidity and mortality were recorded as 100% and 70%, respectively [30]. Similar lesions caused by the DPV infection, as described by Jahan et al. [4] and Ahamed et al. [13], were found in the postmortem examination of ducklings, including widespread pinpoint hemorrhages in the liver. The selected isolate of the virus showed high pathogenicity in ducklings, confirming its suitability as a vaccine seed. Specimens obtained from the infected ducks were processed for DPV re-isolation, which was later confirmed by PCR.

Inactivated DPV bulk was found free from bacterial and fungal contamination after incubation on TSB and blood agar media.

The stability of the DP antigen was measured by determining infectivity titer (EID_50_) on the first day, 3 months, and 6 months, which were 10^7.9^, 10^7.8^, and 10^7.7^,, respectively. The vaccine displayed a typical appearance and viscosity, in accordance with the parameters outlined in the OIE Terrestrial Manual [14].

The formulated inactivated vaccines were found to be safe when injected into test ducks. It also confirms the optimal inactivation of the pathogenic DP virus with 0.2% formalin treatment. Formalin (0.04%–0.12%) has been widely used to inactivate DPV in vaccine production, preserving immunogenicity while ensuring safety [2,25]. In the experimental groups G-A, G-B, G-C, G-D and G-E (Table 1) with no signs or symptoms of disease observed during the 7–14-day period [11, 14].

A microplate ELISA test was conducted to measure the OD value of serum samples collected from both the vaccinated and control groups. Before vaccination, the OD values of G-1, G-2, and G-3 were 0.146, 0.147, and 0.148, respectively, which remained under the cut-off value (0.212). The vaccinated groups (G-1 and G-2) revealed the protective antibody titers were above the cut-off value (0.212) in the case of after the first vaccination, whereas the titers of the control group (G-3) were below the cut-off value (Fig. 5) [10,30,35,36]. The ELISA analysis showed that both inactivated vaccines stimulated an immune response, leading to a noticeable increase in antibody levels by day 14, which continued to rise through day 28 compared to the control group. Administration of a booster dose at 30 days after the first vaccination further revealed the steady rise of antibody titer up to 120 days post-vaccination in both vaccinated groups, where the cut-off value was 0.244, indicating a strong and sustained humoral immune response. A significant increase in antibody OD values (average OD: 0.8339 for G2 and 0.957 for G1) was observed following the booster dose compared to the antibody response by the primary vaccination (average OD: 0.319 for G1 and 0.2875 for G2). These findings indicate the establishment of a robust immune response, attributed to the activation and maturation of adaptive immunity induced by the primary vaccination.

**Figure 4.**
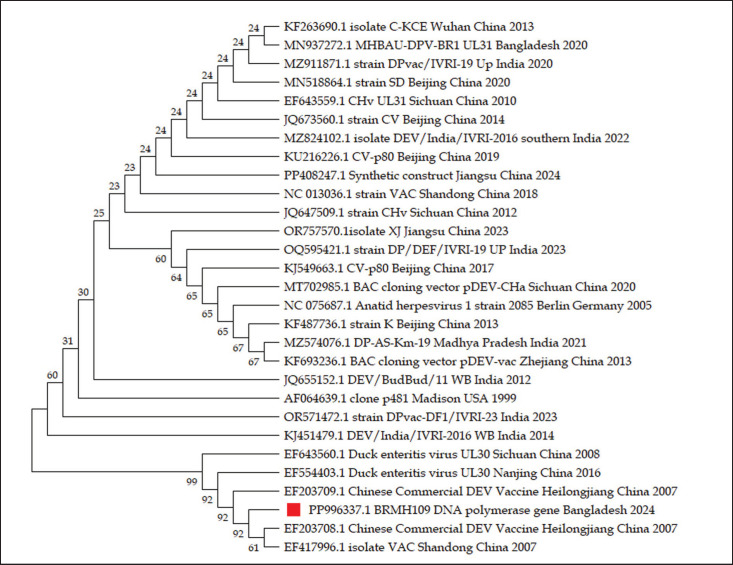
The phylogenetic relationship was constructed from aligned sequences of the partial (346-bp) DNA Polymerase gene of duck plague virus; red marking indicates the BRMH109 isolate.

**Table 1.** Safety test of the two inactivated vaccine groups and the control group.

Group	Age	No. of birds	Type of vaccine	Adjuvant	Healthy	Affected by DPV
G-A	Day-old	05	Inactivated DP vaccine	Montanide ISA 78 VG	05	0
G-B	21 days	05	Inactivated DP vaccine	Montanide ISA 78 VG	05	0
G-C	Day-old	05	Inactivated DP vaccine	Montanide ISA 71 VG	05	0
G-D	21 days	05	Inactivated DP vaccine	Montanide ISA 71 VG	05	0
G-E	21 days	05	Control	1X PBS	05	0

**Figure 5.**
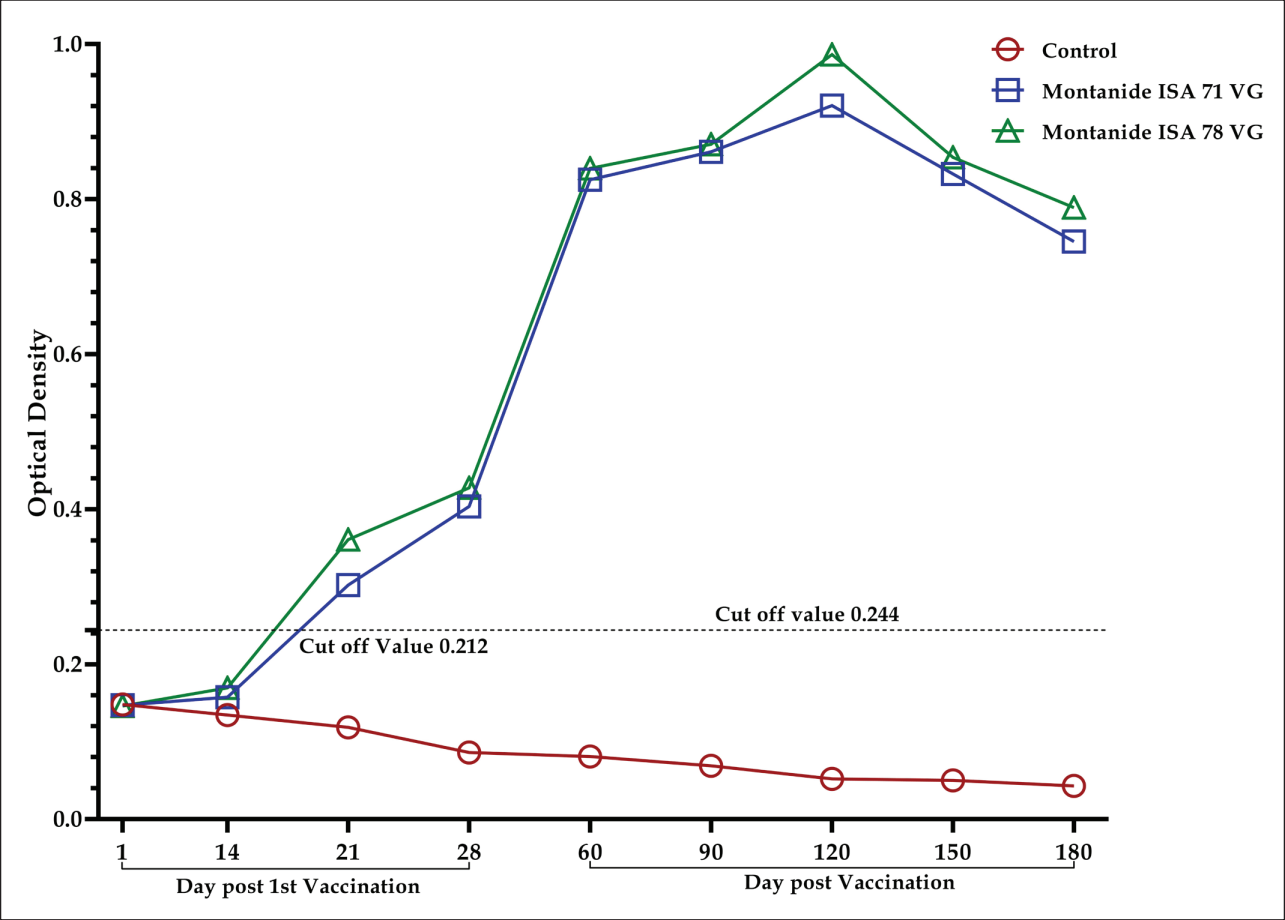
Determination of the antibody titer of the experimental vaccine groups and control group using the ELISA Kit; result interpretation of ELISA by plotting the OD value obtained.

**Table 2.** Challenge test summary of the vaccinated and control groups.

	No. of Ducks	Mortality no.	Mortality		Protection	
			Mortality %	95% Confidence interval	Protection %	95% Confidence interval
Group-1	45	5	11.11	4.84–23.5	88.89	76.5–95.16
Group-2		5	11.11	4.84–23.5	88.89	76.5–95.16
Group-3		42	93.33	82.14–97.71	6.67	2.29–17.86

The ELISA test result confirmed that the experimental inactivated DP vaccine formulated with Montanide ISA 78 VG and 71 VG induced a satisfactory level of antibody titers on the day post-first vaccination, which significantly increased after the booster dose vaccination, as was also described in the previous study [11,33]. The study also indicated a noticeable decline in the antibody titers after 120 days post-vaccination, which continued through 180 days post-vaccination, though it was protective enough. However, the inactivated vaccine demonstrated the ability to provide long-term protection against the DPV infection.

Finally, the efficacy of the vaccine was evaluated by a challenge test. In the challenged study, the inactivated DP vaccine conferred 88.89% protection for both vaccinated groups (G-1 and G-2), while 6.67% was recorded in the G-3 (Table 2) [20,36]. The control group (G-3) exhibited clinical signs and symptoms, including depression, lethargy, and anorexia, and ultimately died from the virulent DPV challenge. The vaccinated group showed protection against field strains of DPV, demonstrating the potential efficacy of the vaccine.

## Conclusion

Duck plague is one of the most economically impactful diseases affecting duck populations. A total of 138 isolates (65.40%) were obtained from 211 samples and confirmed by PCR. The test results revealed that DPV is highly pathogenic for both ducklings and adult ducks. Due to the unavailability of the commercial inactivated duck plague vaccine in the local market, comparative immunogenicity studies using different adjuvants like coral or alum could not be performed. Montanide ISA 78 VG- and 71 VG–based experimentally formulated inactivated DP vaccines were developed and found safe as well as effective against DP infection. Additionally, the study’s findings validated that this vaccine produced a significant level of antibody titers, which could potentially provide 88.89% protection against DP. However, a booster dose of the prepared vaccine was enough to protect the duckling for up to 180 days, exhibiting statistically significant efficacy (*p* < 0.001) and could be considered a viable alternative to the live attenuated duck plague vaccine in Bangladesh. Large-scale field application is possible due to its stability, cost-effective manufacture, and compatibility with the current vaccination programs, especially in endemic zones like the *haor* areas. Widespread vaccination can reduce duck mortality, boost small-scale farmers’ incomes, and improve the flock’s overall health. The use of an inactivated vaccine could improve national duck health management and food security.

## List of abbreviations

CAM, Chorioallantoic Membrane; CPE, Cytopathic Effect; DPV, Duck Plague Virus; EDE, Embryonated Duck Egg; EID, Embryo Infective Dose; ELISA, Enzyme-Linked Immunosorbent Assay; DEF, Duck Embryo Fibroblast; FBS, Fetal Bovine Serum; PHA, Passive Hemagglutination; PBS, Phosphate Buffer Saline; RPM, Rotation Per minute; VTM, Virus Transport Media; µl, microliter; °C, degree Celsius; ml, milliliter.
